# Supplementary material for: Determinants of Influenza Vaccine Uptake Among Rural Populations in a Southeastern U.S. State
Source: Vaccines (Basel). 2025 Nov 29;13(12):1208. doi: 10.3390/vaccines13121208 (PMC12737590; doi:10.3390/vaccines13121208)
Supplement: Supplementary file 1 [file vaccines-13-01208-s001.zip › vaccines-3902362-supplementary S2.pdf]

Table S1. Participant characteristics (Unweighted sample *N* = 421; Weighted sample *N* = 426).

**Variable**	**n (%)**
Sex	
Female	217 (50.9)
Male	209 (49.1)
Race	
White	328 (77.1)
Non-white	98 (22.9)
Ethnicity	
Not Hispanic or Latino	403 (94.6)
Hispanic or Latino	23 (5.4)
Age	
18 – 24	38 (8.9)
25 – 34	68 (15.9)
35 – 44	87 (20.3)
45 – 54	84 (19.7)
55 – 64	65 (15.3)
65+	85 (19.9)
Political affiliation	
Republican	185 (43.5)
Independent	116 (27.3)
Democrat	58 (13.5)
Other	67 (15.7)
Education	
Less than high school	30 (7.1)
High school diploma or graduate equivalency degree	231 (54.2)
Associate degree or vocational certificate	107 (25.0)
4-year bachelor's or higher	59 (13.7)
Household income	
$0–$30,000	165 (38.8)
$30,001–$60,000	129 (30.3)
$60,001–$90,000	71 (16.6)
$90,001–$120,000	25 (5.8)
$120,000+	19 (4.4)
I choose not to say	18 (4.1)
Employment status	
Employed	182 (42.8)
Retired	97 (22.7)
Disabled	59 (13.9)
Not employed	88 (20.6)
Presence of chronic conditions	
Yes	289 (67.8)
No	137 (32.2
Previously received a COVID-19 vaccine in the past	
Yes	206 (48.4)
No	220 (51.6)
Confidence in understanding health information	
High	232 (54.5)
Moderate	140 (32.8)
Low	54 (12.8)

Table S2. Reliability of Trust Scales (*N* =421).

Trust Scale	Components	Number of items	Means (± SD)	Cronbach's alpha
Trust in public health authorities[a]	Beneficence	8	2.55 (.55)	.813
	Competence	6	2.55 (.60)	.797
Trust in doctors in general[b]	Communication competency	5	3.57 (.74)	.857
	Fidelity	5	2.93 (.84)	.859
	Systems trust	3	3.29 (.95)	.885
	Confidentiality	3	3.71 (.83)	.831
	Fairness	7	3.40 (.83)	.925
	Stigma-based discrimination	3	3.17 (.84)	.759
	Global trust	3	3.52 (.92)	.949
Trust in community pharmacists (Trust-Ph)[b]	Benevolence	12	3.32 (.70)	.926
	Technical competence	10	3.46 (.63)	.874
	Communication	8	3.77 (.68)	.918

[a]4-point Likert Scale ranging from strongly disagree to strongly agree; [b]5-point Likert scale ranging from strongly disagree to strongly agree.

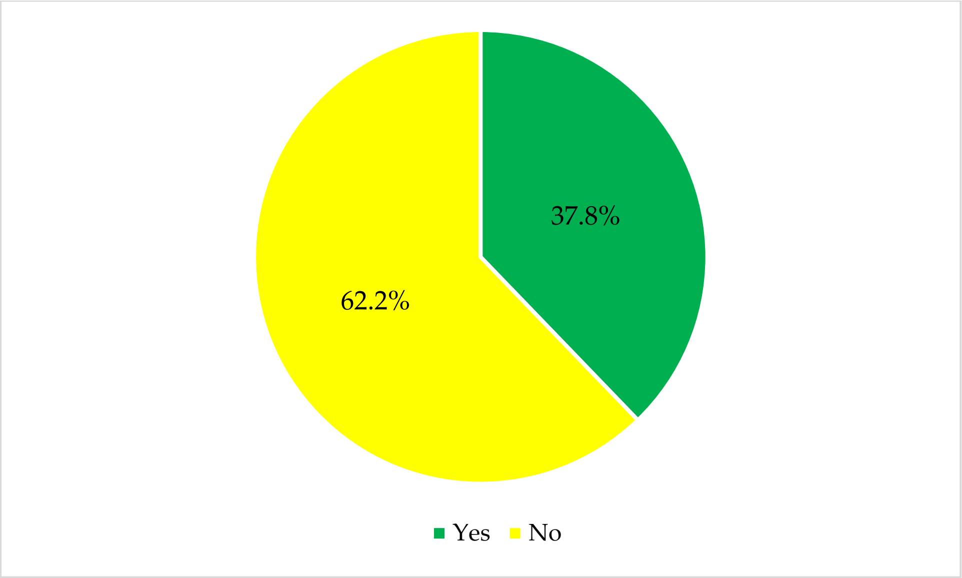

Figure S1. Influenza vaccine uptake ((Unweighted sample *N* = 421; Weighted sample *N* = 426).

Table S3. Bivariate associations between participant characteristics and influenza vaccine uptake in 2023-2024 influenza season (Unweighted sample N = 421; Weighted sample N = 425/6).

Factor[a]	Flu vaccine uptake[a] Yes n = 161 (37.8%) n (%)	No n = 265 (62.2%) n (%)	p – value[b]
Sex			**.020**
Female	70 (43.5)	146 (55.1)	
Male	91 (56.5)	119 (44.9)	
Race			.554
White	121 (75.6)	207 (78.1)	
Non-white	39 (24.4)	58 (21.9)	
Ethnicity			.880
Not Hispanic or Latino	151 (94.4)	251 (94.7)	
Hispanic or Latino	9 (5.6)	14 (5.3)	
Age			**<.001**
18 – 24	13 (8.1)	25 (9.4)	
25 – 34	19 (11.8)	49 (18.4)	
35 – 44	15 (9.3)	72 (27.1)	
45 – 54	23 (14.3)	61 (22.9)	
55 – 64	31 (19.3)	35 (13.2)	
65+	60 (37.3)	24 (9.0)	
Political affiliation			**<.001**
Republican	67 (41.6)	118 (44.5)	
Independent	49 (30.4)	67 (25.3)	
Democrat	33(20.5)	25 (9.4)	
Other	12 (7.5)	55 (20.8)	
Education			**<.001**
Less than high school	5 (3.1)	25 (9.4)	
High school diploma or graduate equivalency degree	64 (39.8)	167 (62.8)	
Associate degree or vocational certificate	60 (37.3)	47 (17.7)	
4-year bachelor's or higher	32 (19.9)	27 (10.2)	
Household income			**0.006**
$0–$30,000	47 (29.2)	119 (44.7)	
$30,001–$60,000	53 (32.9)	76 (28.6)	
$60,001–$90,000	31 (19.3)	39 (14.7)	
$90,001–$120,000	13 (8.1)	12 (4.5)	
$120,000+	12 (7.5)	7 (2.6)	
I choose not to say	5 (3.1)	13 (4.9)	
Employment status[a]			**<.001**
Employed	60 (37.3)	122 (46.0)	
Retired	63 (39.1)	34 (12.8)	
Disabled	24 (14.9)	35 (13.2)	
Not employed	14 (8.7)	74 (27.9)	
Presence of chronic conditions			**.002**
Yes	124 (77.0)	165 (62.3)	
No	37 (23.0)	100 (37.7)	
Previously received a COVID-19 vaccine in the past[a]			**<.001**
Yes	137 (85.1)	69 (26.0)	
No	24 (14.9)	196 (74.0)	
Confidence in understanding health information			.973
High	88 (54.7)	144 (54.3)	
Moderate	52 (32.3)	88 (33.2)	
Low	21 (13.0)	33 (12.5)	

[a]Chisquare; [b]bold = p < .05 / significant.

Table S4. A binary logistic regression analysis of participants' trust in medical doctors, pharmacists, and public health authorities on flu vaccine uptake (Unweighted sample *N* = 421; Weighted sample *N* = 426).

Trust scale	Odds ratio[a]	95% confidence interval		*P* value[b]
		Lower	Upper	
Trust in Public Health				
Beneficence	2.291	**1.553**	**3.378**	**<.001**
Competence	2.200	**1.544**	**3.133**	**<.001**
Trust in doctors in general				
Communication competency	2.444	**1.781**	**3.352**	**<.001**
Fidelity	1.599	**1.255**	**2.037**	**<.001**
Systems trust	1.414	**1.140**	**1.753**	**.002**
Confidentiality	1.336	**1.047**	**1.705**	**.020**
Fairness	1.381	**1.083**	**1.761**	**.009**
Stigma-based discrimination	1.434	**1.129**	**1.821**	**.003**
Global trust	1.841	**1.442**	**2.351**	**<.001**
Trust in Community Pharmacists				
Benevolence	1.634	**1.213**	**2.200**	**.001**
Technical competence	1.403	**1.016**	**1.937**	**.039**
Communication	1.369	**1.015**	**1.846**	**.040**

[a]Reference category is no flu uptake; [b]bold = $p < .05$ / significant.

Table S5. Multicollinearity results for significant predictors identified in the bivariate analyses (Unweighted sample *N* = 421; Weighted sample *N* = 426).

Variable.	Tolerance	Variance Inflation Factor (VIF)
Sex	.891	1.122
Age	.555	1.801
Political affiliation	.871	1.148
Education	.810	1.235
Household income	.907	1.103
Employment	.728	1.374
Presence of chronic conditions	.885	1.130
Previously received a COVID-19 vaccine in the past	.658	1.521
Trust in Public Health		
Beneficence	.279	3.583
Competence	.279	3.590
Trust in doctors in general		
Communication competency	.407	2.454
Fidelity	.585	1.708
Systems trust	.488	2.049
Confidentiality	.478	2.093
Fairness	.403	2.482
Stigma-based discrimination	.724	1.380
Global trust	.400	2.500
Trust in Community Pharmacists		
Benevolence	.282	3.546
Technical competence	.301	3.324
Communication	.360	2.779

Table S6. A multivariable logistic regression of factors associated with flu vaccine uptake (Unweighted sample *N* = 421; Weighted sample *N* = 426).

Factor	Adjusted Odds ratio[a]	95% confidence interval		*P* value[b]
		Lower	Upper	
Sex (Ref = male)				
Female	.809	.438	1.493	.498
Age (Ref = 18 –24)				
25 – 34	.901	.260	3.124	.869
35 – 44	.551	.161	1.883	.342
45 – 54	.525	.158	1.751	.295
55 – 64	2.258	.593	8.596	.233
65+	2.531	.569	11.257	.223
Political affiliation (Ref = republican)				
Independent	2.142	.984	4.664	.055
Democrat	2.504	**1.017**	**6.164**	**.046**
Other	.692	.251	1.906	.476
Education (Ref = less than high school)				
High school diploma or graduate equivalency degree	.705	.196	2.544	.594
Associate degree or vocational certificate	1.837	.458	7.370	.391
4-year bachelor's or higher	.973	.220	4.303	.971
Income (Ref = $0–$30,000)				
$30,001–$60,000	1.723	.854	3.474	.128
$60,001–$90,000	1.194	.489	2.916	.697
$90,001–$120,000	1.164	.342	3.964	.809
$120,000+	6.172	**1.538**	**24.769**	**.010**
I choose not to say	1.220	.255	5.840	.804
Employment (Ref = employed)				
Retired	1.429	.548	3.727	.466
Disabled	2.571	.974	6.785	.057
Not employed	.672	.285	1.582	.362
Presence of chronic conditions (Ref = yes)				
no	1.370	.711	2.643	.347
Previously received a COVID-19 vaccine in the past (Ref = no)				
Yes	9.790	**4.923**	**19.467**	**<.001**
Trust in Public Health				
Beneficence	.782	.303	2.019	.611
Competence	1.722	.714	4.154	.226
Trust in doctors in general				
Communication competency	3.090	**1.560**	**6.118**	**.001**
Fidelity	1.088	.688	1.718	.719
Systems trust	1.295	.852	1.967	.227
Confidentiality	.973	.578	1.638	.919
Fairness	.589	.344	1.010	.055
Stigma-based discrimination	1.046	.701	1.561	.824
Global trust	.812	.479	1.375	.438
Trust in Community Pharmacists				
Benevolence	1.152	.530	2.504	.721
Technical competence	.983	.438	2.205	.966
Communication	.601	.300	1.204	.151

[a]Reference category is no flu uptake; [b]bold = $p < .05$ / significant.

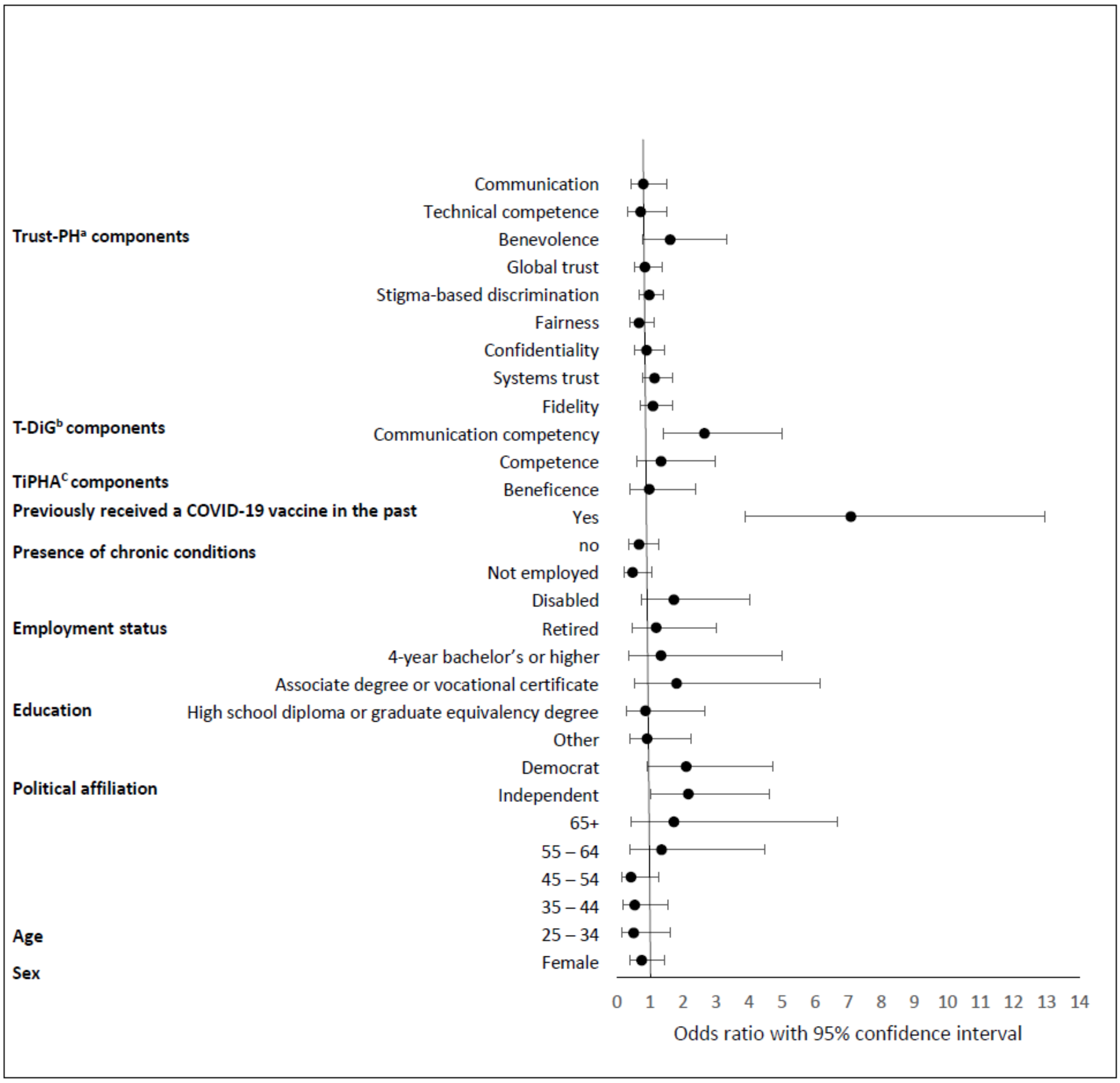

Figure S2. A multivariable logistic regression of factors associated with flu vaccine uptake (*N* = 421). aTrust in Community Pharmacists scale; b Trust in Doctors in General scale; cTrust in Public Health Authorities scale. Reference categories: Sex (male), age (18 -24 years), political affiliation (republican), education (less than high school), employment (employed), presence of chronic conditions (yes), Previously received a COVID-19 vaccine in the past (no).

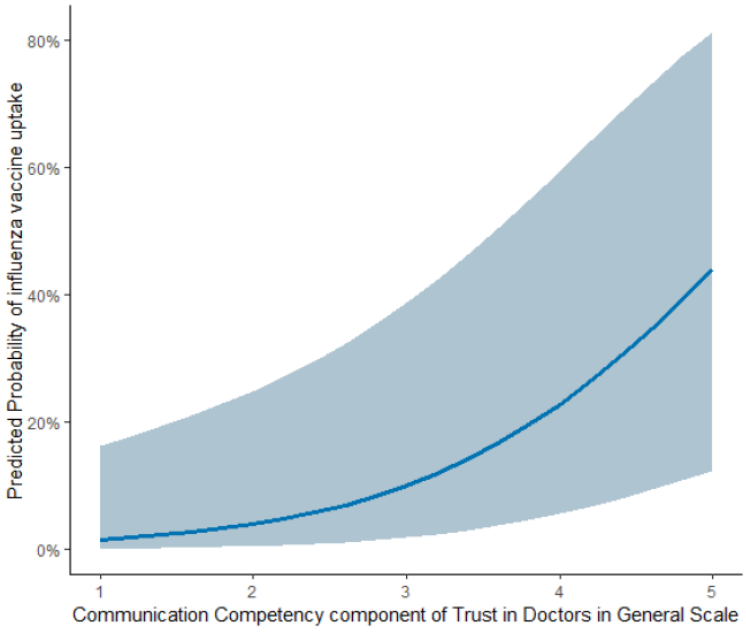

Figure S3. Marginal effects plot of communication competency on influenza vaccine uptake (*N* = 421).

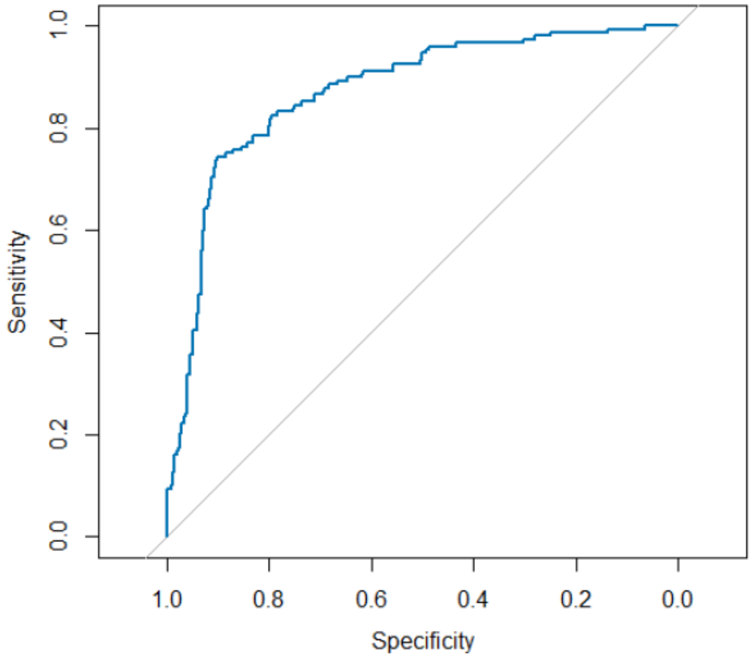

Figure S4. Receiver operating characteristic (ROC) curve for influenza vaccine uptake model (*N*= 421).

Table S7. Bootstrap Validation of the Multivariable Logistic Regression Model.

Metric	Value
Original Estimate	0.829
Bootstrap Bias	0.012
Standard Error	0.021
Resamples	1,000
Method	Ordinary Nonparametric Bootstrap

Table S8. Linearity diagnostics for continuous predictors in the multivariable logistic regression model using Box-Tidwell test (N= 421).

Factor and their interactions	Estimate	Standard error	p value
Trust in Public Health Authorities			
Beneficence	-3.692	3.317	0.266
Beneficence x log (Beneficence)	2.057	1.721	0.232
Competence	1.990	3.199	0.534
Competence x log (Competence)	-0.869	1.657	0.600
Trust in doctors in general			
Communication competency	2.645	3.236	0.414
Communication competency x log (Communication competency)	-0.772	1.437	0.591
Fidelity	2.463	1.996	0.217
Fidelity x log (Fidelity)	-1.145	0.942	0.224
Systems trust	0.720	1.722	0.676
Systems trust x log (Systems trust)	-0.324	0.801	0.686
Confidentiality	0.302	2.384	0.899
Confidentiality x log (Confidentiality)	-0.228	1.048	0.828
Fairness	1.975	2.313	0.393
Fairness x log (Fairness)	-1.012	1.053	0.337
Stigma-based discrimination	-1.773	1.803	0.325
Stigma-based discrimination x log (Stigma-based discrimination)	0.919	0.862	0.286
Global trust	-1.105	1.860	0.552
Global trust x log (Global trust)	0.568	0.859	0.509
Trust in Community Pharmacists			
Benevolence	2.813	3.055	0.357
Benevolence x log (Benevolence)	-1.136	1.399	0.417
Technical competence	-1.250	3.555	0.725
Technical competence x log (Technical competence)	0.292	1.615	0.856
Communication	-4.358	2.975	0.143
Communication x log (Communication)	1.894	1.324	0.152

Table S9. Post-hoc pairwise contrasts of political affiliation effects adjusted for multiple comparisons in the multivariable logistic regression model (n= 421).

Contrast	estimate	Standard error	Odds ratio	Lower 95% CI	Upper 95% CI	p value
Republican- Democrat	-0.744	0.413	0.475	0.160	1.414	0.431
Republican - Independent	-0.776	0.384	0.460	0.167	1.267	0.259
Republican - Other	0.079	0.452	1.082	0.328	3.568	1.000
Democrat - Independent	-0.033	0.445	0.968	0.299	3.130	1.000
Democrat - Other	0.823	0.514	2.277	0.587	8.831	0.656
Independent - Other	0.856	0.483	2.353	0.659	8.403	0.457

p-values were adjusted using the Bonferroni method to control the family-wise error rate across six pairwise comparisons of political affiliation groups. The adjusted significance threshold was set at $\alpha = 0.05 / 6 = 0.0083$.
